# Transcriptomic analysis of the stationary phase response regulator SpdR in *Caulobacter crescentus*

**DOI:** 10.1186/s12866-016-0682-y

**Published:** 2016-04-12

**Authors:** Carolina A. P. T. da Silva, Rogério F. Lourenço, Ricardo R. Mazzon, Rodolfo A. Ribeiro, Marilis V. Marques

**Affiliations:** Departamento de Microbiologia, Instituto de Ciências Biomédicas, Universidade de São Paulo, Av. Prof. Lineu Prestes 1374, 05508-000 São Paulo, SP Brazil; Departamento de Bioquímica, Instituto de Química, Universidade de São Paulo, Av. Prof. Lineu Prestes 748, 05508-000 São Paulo, SP Brazil; Present address: Departamento de Microbiologia, Imunologia e Parasitologia, Centro de Ciências Biológicas, Universidade Federal de Santa Catarina, Campus Universitário da Trindade, Caixa postal 476, 88040-900 Florianópolis, SC Brazil

**Keywords:** Stationary phase, Transcriptional regulation, Two-component system, *Caulobacter*

## Abstract

**Background:**

As bacterial cells enter stationary phase, they adjust their growth rate to comply with nutrient restriction and acquire increased resistance to several stresses. These events are regulated by controlling gene expression at this phase, changing the mode of exponential growth into that of growth arrest, and increasing the expression of proteins involved in stress resistance. The two-component system SpdR/SpdS is required for the activation of transcription of the *Caulobacter crescentus cspD* gene at the onset of stationary phase.

**Results:**

In this work, we showed that both SpdR and SpdS are also induced upon entry into stationary phase, and this induction is partly mediated by ppGpp and it is not auto-regulated. Global transcriptional analysis at early stationary phase of a *spdR* null mutant strain compared to the wild type strain was carried out by DNA microarray. Twenty-three genes showed at least twofold decreased expression in the *spdR* deletion mutant strain relative to its parental strain, including *cspD*, while five genes showed increased expression in the mutant. The expression of a set of nine genes was evaluated by quantitative real time PCR, validating the microarray data, and indicating an important role for SpdR at stationary phase. Several of the differentially expressed genes can be involved in modulating gene expression, including four transcriptional regulators, and the RNA regulatory protein Hfq. The ribosomal proteins NusE and NusG, which also have additional regulatory functions in transcription and translation, were also downregulated in the *spdR* mutant, as well as the ParE1 toxin. The purified SpdR protein was shown to bind to the regulatory region of CC0517 by Electrophoretic Mobility Shift Assay, and the SpdR-regulated gene CC0731 was shown to be expressed at a lower level in the null *cspD* mutant, suggesting that at least part of the effect of SpdR on the expression of this gene is indirect.

**Conclusions:**

The results indicate that SpdR regulates several genes encoding proteins of regulatory function, which in turn may be required for the expression of other genes important for the transition to stationary phase.

**Electronic supplementary material:**

The online version of this article (doi:10.1186/s12866-016-0682-y) contains supplementary material, which is available to authorized users.

## Background

The fundamental characteristic of bacterial cells is the ability to regulate their growth in response to environmental changes. The stationary phase in bacteria is characterized by growth arrest in response to several external factors, such as nutrient starvation, accumulation of toxic compounds and environmental stresses. Bacteria utilize varied mechanisms for coping with these situations, but the main effect is the decrease of ribosome activity, resulting in a great reduction in protein synthesis. In order to maintain viability during growth arrest, cells need to reorganize their metabolism, using several regulatory factors to define new protein expression profiles. Proteins produced by cells at the onset of stationary phase are involved in their survival through long periods of nutrient starvation, maintaining only essential cellular functions [1, 2]. In addition, cells acquire greater resistance to stress conditions, including cold shock, oxidative stress and osmotic variations [3].

Changes in gene expression during the transition from exponential to stationary phase respond primarily to the nutritional status of the cell. In Enterobacteria, where response to stationary phase has been well characterized, the major global regulator of this response is the alternative sigma factor σ^S^, which directs transcription of genes involved in stress response, as well as metabolic functions and uptake and metabolism of amino acids, sugars and metals [4]. Transcriptional response to stationary phase is also mediated by the signaling molecule guanosine tetraphosphate (ppGpp), which binds to RNA polymerase core, destabilizing its association to strong promoters of rDNA genes and therefore releasing the core enzyme for transcription of specific genes [5].

*Caulobacter crescentus* is an alphaproteobacterium that grows in low-nutrient aquatic environments [6]. After entry into stationary phase, the majority of *C. crescentus* cells stays in the predivisional-stage and gradually acquires a helicoidal, elongated morphology, with increased stress resistance compared to exponentially growing cells [7]. Transcriptional response and gene regulation during stationary phase is still poorly documented in *C. crescentus*. Currently, three alternative extracytoplasmic function sigma factors, namely σ^F^, σ^T^, and σ^U^, are known to mediate the bacterial response to stationary phase [8, 9]. Noticeably, σ^T^ has been proposed to be the master regulator of general stress response in *C. crescentus*, therefore playing analogous function of *E. coli* σ^S^ [9, 10]. Furthermore, some small regulatory RNAs are induced in stationary phase by nutrient starvation, suggesting that the regulatory network that controls gene expression in this phase is much more complex [11]. Likewise, the contribution of specific stationary phase-induced genes for *C. crescentus* adaptation to this growth phase is largely unknown, being mostly limited to *katG*, which encodes a catalase-peroxidase, *cspC* and *cspD,* coding for cold shock proteins [12–16]. *cspD* encodes a protein of the Cold Shock family containing two Cold Shock Domains, which is induced upon entry into stationary phase [14]. The Cold Shock Domain is composed of two RNP1 sequence motifs that were demonstrated to bind nucleic acids [17]. The CspD protein was implicated in repressing DNA replication in *E. coli* [18] and it is regulated both transcriptionally [19] and by proteolysis [20] in this bacterium.

Previously, the response regulator SpdR belonging to the two-component system SpdR/SpdS was characterized in *C. crescentus* by its ability to directly bind the promoter region of the *cspD* gene and activate its transcription at stationary phase [21]. A conserved aspartic acid residue at position 64 of SpdR is essential for SpdR binding to the *cspD* promoter, and it was proposed to be the site of phosphorylation by its cognate histidine kinase SpdS. SpdS possesses a transmembrane segment that separates an extracytoplasmic sensor domain from the cytoplasmic autophosphorylation/phosphate transfer domain. In this work, we have characterized the SpdR regulon, providing a start point for understanding how this regulator mediates the adaptation to stationary phase in *C. crescentus.* We demonstrate that SpdR and SpdS are induced upon entry into stationary phase, and that several genes regulated by this two-component system are involved in adjusting the overall gene expression rate to ensure adaptation to this phase.

## Methods

### Bacterial strains and growth conditions

*C. crescentus* and *E. coli* strains, as well as the plasmids utilized in this work, are listed in Table S1 (Additional file 1). *C. crescentus* NA1000 and derived strains were grown at 30 °C in PYE or M2 medium [22]. The media were supplemented with tetracycline (1 μg/ml) for growing strains harboring pRK*lacZ*290 and kanamycin (5 μg/ml) for strains harboring pNPTS138. *Escherichia coli* DH5α and BL-21 were used for cloning procedures and protein expression, respectively, and were grown at 37 °C in Luria-Bertani medium [23] supplemented with ampicillin (100 μg/ml), tetracycline (12.5 μg/ml) and kanamycin (50 μg/ml) as needed. None of the bacterial strains used in this study required ethical approval to use.

### Heterologous expression of His-SpdR and mouse immunization

The coding region (558 bp) of the *spdR* gene was amplified by PCR using oligonucleotides REG-1 and REG-2. The resulting fragment was cloned in vector pET28a in order to express the SpdR protein with a histidine tag (His_6_-SpdR) in *E. coli* BL-21, and protein expression was induced at 37 °C in the presence of 300 μM IPTG. Purification of His_6_-SpdR was carried out using a Ni-affinity column chromatography according to the manufacturer’s instructions (Qiagen).

Ten 6-week-old male SPF Balb/c mice were kept five animals/isolator in a free water and food regimen, in a 12 h light/dark cycle, with room temperature at 22 °C. The mice were immunized with four weekly injections of 20 μg purified His_6_-SpdR and 50 μl Freund’s adjuvant in a total of 100 μl each injection, during 4 weeks. The first immunization was subcutaneous and contained Freund’s complete adjuvant, and the subsequent immunizations were intraperitoneal and contained Freund’s incomplete adjuvant. One week after the last immunization, animals were anesthetized with 80 mg/kg ketamine and 10 mg/kg xilazin (União Química Farmacêutica, Brazil), and blood was collected by cardiac puncture. Immune sera of 10/10 animals were combined, and tested for specificity in immunoblots. All procedures were approved by the Biomedical Sciences Institute Ethics Committee (Protocol Register 037), and follow the Ethical Principles for Animal Experimentation of the Brazilian Society of Laboratory Animals Science. This work adheres to ARRIVE guidelines (Additional file 2).

### Immunoblots

*C. crescentus* strains NA1000 and ∆*spdR* were grown at 30 °C in PYE medium and proteins were extracted both at exponential (OD_600_ = 0.5) and stationary (24 h) phases. Aliquots (1 ml) were centrifuged for 5 min and cells were suspended in Laemmli’s sample buffer. The volume of buffer was calculated according to the optical density of cultures to ensure similar protein concentrations. Proteins were separated in 15 % SDS-polyacrylamide gels, using PAGE Ruler Prestained Protein Ladder (Fermentas) as molecular weight marker. After electrophoresis, proteins were transferred to nitrocellulose filters and immunoblots were carried out as previously described [24]. Briefly, nitrocellulose filters were incubated with mild agitation for 1 h in TBS (10 mM Tris-Cl pH 8.0, 150 mM NaCl) containing 5 % nonfat milk, and then incubated for approximately 16 h with diluted anti-serum (1:50) in TBSTT (TBS with 0.03 % Tween 20, 0.02 % Triton X-100). Filters were incubated for 2 h at room temperature with anti-mouse antibody conjugated with alkaline phosphatase (Sigma), diluted 1:5000 in TBS, followed by color development with 0.5 mg/ml NBT and 0.15 mg/ml BCIP in alkaline phosphate buffer (100 mM Tris-Cl pH 9.5, 5 mM MgCl_2_, 100 mM NaCl).

### Construction of vector pCA60 and analysis of *spdS* promoter activity

A PCR using oligonucleotides AUTO-1 and HIST-1 was carried out to amplify a 400 bp fragment (from −1 to −400 relative to the annotated translational start site of *spdS*). The fragment was cloned into vector pRK*lacZ*290 previously digested with enzymes EcoRI and BamHI and the resulting construction (pCA60) was introduced into *E. coli* S17-1 by electroporation, and transferred by conjugation to *C. crescentus* strains NA1000, Δ*spdR* and Δ*spoT*.

Cultures containing plasmid pCA60 were diluted to an OD_600_ = 0.1 and promoter activity was assessed by β-galactosidase activity assays [25] in both exponential (OD_600_ = 0.5) and stationary phases (OD_600_ = 1.2–1.3, 24 h after dilution). All experiments were performed in duplicates from three biological replicates.

### DNA microarrays

Cultures of both the parental NA1000 and Δ*spdR* strains were grown up to early stationary phase (24 h growth, OD_600_ = 1.2–1.3) in PYE medium. Total RNA was extracted from 10 ml-cultures with Trizol reagent (Invitrogen) as instructed by the manufacturer. RNA was quantified with NanoDrop 2000 (Thermo Scientific) and 50 μg of each sample were treated with 25 units of DNAse I (Fermentas). Absence of DNA was confirmed by PCR. cDNA was generated with the FairPlay III Microarray Labeling Kit (Agilent Technologies), and 24 μg of each sample were purified and precipitated. The Cy3 (Q13108, GE Healthcare) and Cy5 (Q15108, GE Healthcare) Monofunctional Reactive Dyes were used to label NA1000 and Δ*spdR* samples, respectively. Fluorophores coupling to cDNA was performed in the buffer supplied by the manufacturer, and labeled and purified cDNAs were quantified with NanoDrop 2000.

Each hybridization reaction (NA1000 x Δ*spdR*) was mounted on 4x44K microarray slides customized for *Caulobacter* (Agilent Technologies), with the same amount of cDNA for each sample, and slides were incubated for 24 h at 65 °C and 10 rpm. Fluorescence on slides was scanned using the SureScan Microarray Scanner (Agilent Technologies), and values of relative expression were obtained through the Feature Extraction Software (Agilent Technologies). The customized slides for *Caulobacter* include oligonucleotides that hybridize with non-coding regions of the genome; since only coding regions were of our interest in this work, we analyzed only the four last oligonucleotides of a given ORF, which mapped inside the open reading frame. In order to be considered down- or upregulated, a gene must have displayed at least three out of the four last oligonucleotides with Cy5/Cy3 ratio values (mutant/NA1000) below 0.5 (downregulated) or above 2 (upregulated) in at least three out of the four biological replicates. Cy5/Cy3 ratio values from the last four oligonucleotides of all replicates were averaged.

### Quantitative RT-PCR (qRT-PCR)

Stationary phase RNA samples (12 μg) of strains NA1000 and Δ*spdR* were treated with six units of DNAse I (Fermentas), and approximately 3 μg were used as template for cDNA synthesis. Real-time PCR was performed using 50 ng cDNA, 0.1 μM oligonucleotides specific for each gene, and the Maxima SYBR Green/ROX qPCR Master Mix (Fermentas). Fluorescence emitted was analyzed with the 7500 System SDS Software v.1.2.2 (Applied Biosystems). All oligonucleotides used for this analysis (Additional file 1: Table S1) were designed with the Primer-BLAST software [26] and displayed equivalent amplification efficiency. The 2^-ΔΔCT^ method [27, 28] was utilized to calculate relative expression of genes, with ORF CC3098 as normalizer.

### Identification of possible regulatory sequences

Genes identified in the microarray experiments were analyzed in a search for a putative SpdR-binding motif using the “DNA-pattern” module of RSAT (Regulatory Sequence Analysis Tools, available at http://www.rsat.eu/) [29]. The sequence CTGCGAC-N_5_-GTCGCGG, previously found to be directly recognized by SpdR [21], and the sequences better matching to a perfect palindromic motif (CTGCGAC-N_5_-GTCGCAG and CCGCGAC-N_5_-GTCGCGG) were utilized as template and up to two substitutions were allowed.

### Electrophoretic Mobility Shift Assays (EMSA)

Promoter regions of genes CC0517 and CC1746 were amplified by PCR with the oligonucleotide pairs SHIFT CC0517 Forward/Reverse and SHIFT CC1746 Forward/Reverse respectively. Probes were end-labeled with 20 μCi [γ-^32^P] ATP using T4 polynucleotide kinase (Invitrogen), and DNA-binding reactions were performed in a volume of 30 μl containing 0, 25, 50, 100, 250 or 500 nM purified SpdR protein. In competition assays, a 30x excess of unlabeled specific fragment (specific competitor) was added to the labeled specific fragment; in another reaction, a 30x excess of an unlabeled fragment containing the *cspD* coding region (non-specific competitor) was used together with the labeled specific fragment. Both reactions were carried out with 50 nM purified SpdR. After incubation at 30 °C for 30 min, samples were run in a 5 % polyacrylamide gel in 0.5X TBE buffer, the gel was subsequently dried and exposed to an X-ray film.

### Construction of the mutant strains

For obtaining a ΔCC0517 mutant strain, the flanking regions of gene CC0517 were amplified by PCR using the oligonucleotide pairs HIP-1/HIP-2 and HIP-3/HIP-4, and for the Δ*spdR* mutant, the flanking regions of gene CC0247 were amplified by PCR using pairs RR1/RR2 and RR3/RR4. The respective fragments were cloned *in tandem* into pNPTS138, a suicide vector in *C. crescentus*, and the resulting recombinant plasmids were introduced into *E. coli* S17-1 and subsequently in *C. crescentus* NA1000 by conjugation, generating strains MM80 (Δ*CC0517*) and MM85 (Δ*spdR*) after double recombination.

### Statistical methods

Statistical analysis was performed using Students’ *T*-test, and p values <0.05 were considered significant.

## Results and discussion

### SpdR and SpdS induction at stationary phase

**Fig. 1:**
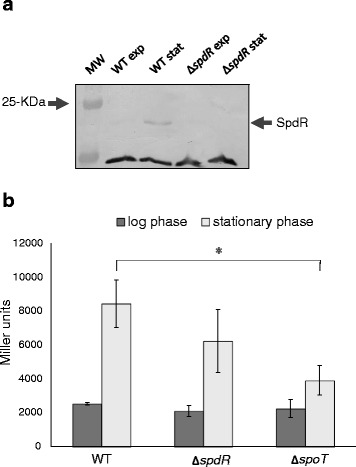
Analysis of SpdR and SpdS expression at stationary phase. **a** Total proteins from NA1000 and *spdR* mutant strains were extracted in exponential phase (exp) and stationary phase (stat) in PYE medium and separated by a 12 % SDS-PAGE. Following protein transfer to a nitrocellulose filter, an immunoblot assay was carried out with anti-SpdR anti-serum (1:500), identifying the 21-kDa band corresponding to SpdR. The 25-kDa band of the prestained Molecular Weight marker (MW) is indicated. A non-specific band recognized by the antiserum is shown to allow assessment of the protein concentration in each lane. **b** The expression driven by the *spdS* promoter cloned upstream of the *lacZ* reporter gene was assessed by β-galactosidase activity assays, both in logarithmic and stationary phases. The reporter plasmid was introduced into the *C. crescentus* strains NA1000, MM85 (Δ*spdR*) and SP0200 (Δ*spoT*). Results are the means of three experiments, and bars indicate the respective standard errors. Asterisk indicates a statistical difference between the parental strain and ∆*spoT* strain at stationary phase (*p* < 0.01) as determined by Students’ *T*-test

As the SpdR-regulated gene *cspD* is induced following *C. crescentus* entry into stationary phase [21], we firstly investigated whether this induction is consequence of an increase in expression of its regulatory protein SpdR at the same growth phase. A polyclonal antiserum raised in mice against His-SpdR was able to recognize the protein in immunoblots. According to immunoblotting assays with the anti-SpdR serum, the levels of SpdR in exponentially growing cells are virtually undetected, but increase dramatically at stationary phase (Fig. 1a). The control lanes show that the protein was absent in the *spdR* deletion strain. As *spdR* and its cognate histidine kinase gene *spdS* were described as not belonging to the same transcriptional unit [30], we further measured *spdS* expression by assaying the promoter activity of the region upstream of *spdS* in a *lacZ* transcriptional fusion. According to this analysis, *spdS* expression also increases during stationary phase, and this induction was found to be independent of SpdR, since the difference observed was not statistically significant (Fig. 1b). Nonetheless, *spdS* induction is partially compromised in a *spoT* null mutant relative to the parental strain, indicating that ppGpp plays a role in regulating this gene, as observed previously for the *spdR* gene [21].

Previous cDNA microarray studies revealed that SpdR expression varies in response to distinct cultivation conditions. Expression of *spdR* was higher in M2 medium supplemented with xylose in relation to PYE medium (2.33 fold higher) and M2 containing glucose (1.66 fold higher) [31]. It has also been observed that *spdR* is induced under conditions of carbon starvation [32] and that the SpdR/SpdS two-component system was induced under chromate and dichromate stress [33]. These reports and the results from this work suggest that *C. crescentus* SpdR acts on the regulation of target genes to respond to environmental clues that indicate nutrient starvation and stress.

The results indicate that *spdS* and *spdR* expression at stationary phase is a result of transcriptional regulation partly mediated by ppGpp, and *spdS* induction is not dependent on SpdR. Since this transduction system probably works by conveying a signal that results in the phosphorylation of SpdR and its activation, the increase in the concentration of these proteins should amplify the resulting effect on gene regulation.

### Determination of the SpdR regulon

With the aim of identifying additional genes to *cspD* under the control of SpdR, we compared the transcriptional profile of the wild type strain with that of the *spdR* deletion mutant by DNA microarray experiments. As SpdR is induced at stationary phase and is also necessary for the increase of expression of *cspD* that happens at this phase, the comparison was carried out with early stationary phase RNA samples. According to this analysis, expression of *spdR* itself, *cspD* and 22 additional genes were at least twofold lower in the *spdR* deletion mutant strain relative to its parental strain (Table 1). Additionally, this comparison showed that an *spdR* deletion increased the transcript levels of five genes, which have been predicted to encode mostly proteins of unknown function. Interestingly, among these genes, seven were predicted to be essential (CC0653, CC0035, CC0260, CC1247, CC1745, CC2912 were downregulated and CC3655 was upregulated) [34].

**Table 1:** Genes differentially expressed in the Δ*spdR* mutant relative to the wild type strain

Gene^a^	Fold change^b^	Putative function^c^
Downregulated		
CC0035	0.229	Small subunit ribosomal protein S15
CC0247	0.163	Two-component system, response regulator SpdR
CC0260	0.483	Ribonucleoside-diphosphate reductase beta chain
CC0445	0.366	GntR family transcriptional regulator NagR
CC0446	0.231	TonB-dependent receptor NagA
CC0482	0.327	5-methyltetrahydropteroyltriglutamate/homocysteine S-methyltransferase
CC0517	0.289	Protein of unknown function
CC0583	0.380	Succinylarginine dihydrolase
CC0653	0.331	CarD_CdnL_TRCF family transcriptional regulator
CC0679	0.380	Abi-domain protein
CC0731	0.340	Protein of unknown function
CC0873	0.385	Toxin ParE1 from a toxin-antitoxin system
CC1005	0.354	Protein of unknown function
CC1247	0.317	Small subunit ribosomal protein S10/NusE
CC1363	0.456	Membrane-bound proton translocating pyrophosphatase
CC1387	0.344	Cold-shock protein CspD
CC1745	0.291	RNA-binding protein Hfq
CC1746	0.312	GTP-binding protein HflX
CC1991	0.470	Preprotein translocase subunit SecD
CC2912	0.350	Quinolinate synthetase
CC3164	0.389	Cro/CI family transcriptional regulator
CC3205	0.456	Transcription antitermination protein NusG
CC3268	0.455	Protein of unknown function
CC3270	0.394	Cro/CI family transcriptional regulator
Upregulated		
CC2114	2.331	Methyltransferase of unknown specificity
CC2234	2.924	Protein of unknown function
CC3404	2.740	Protein of unknown function
CC3654	29.412	Protein of unknown function
CC3655	17.857	Malate dehydrogenase

^*a*^According to the Kyoto Encyclopedia of Genes and Genomes (KEGG) database for the *C. crescentus* CB15 genome

^*b*^Values are the ∆*spdR*/WT ratio determined by microarray hybridization of RNA samples isolated from cells at the stationary growth phase (24 h after dilution of culture to OD_600_ = 0.1). Genes with *M* value of < 0.5 or > 2.0 were assumed as differentially expressed between strains analyzed. Results shown are the average of four independent biological experiments

^*c*^According to a reanalysis of the deduced protein sequences by using Pfam [61] and BLASTP [62] to search for conserved domains and proteins with predicted function, respectively

**Fig. 2:**
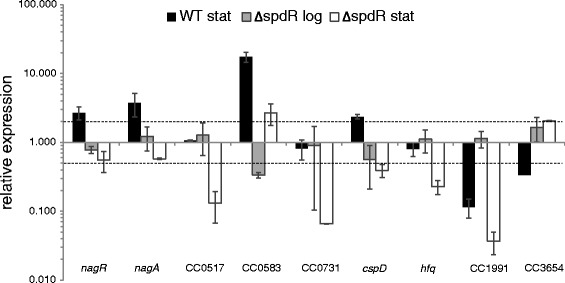
Relative expression of SpdR-regulated genes. Expression of the indicated genes was analyzed by qRT-PCR using total RNA samples obtained from the wild type NA1000 and the ∆*spdR* strain at both exponential and stationary phases. Results represent the expression of each gene in the corresponding strain and growth phase relative to exponentially growing wild type cells. Data represent mean values from two biological replicates, with bars indicating the standard errors

A total of nine genes (eight downregulated genes, and one upregulated gene) were selected for expression analysis by quantitative real-time PCR (qRT-PCR) (Fig. 2). Accordingly, all the genes analyzed displayed altered expression in the *spdR* mutant with respect to the wild type when expression was monitored in cells at stationary phase, thus validating the global approach employed to identify SpdR target genes. When the comparison was performed with samples taken from exponentially growing cells, only expression of CC0583 was changed in the absence of *spdR*. Nonetheless, the fold change in CC0583 expression was still more pronounced at stationary phase. Together, these results are in agreement with the induction of both SpdR and SpdS in the wild type strain at stationary phase, and suggest that regulatory system(s) other than SpdS-SpdR contribute(s) to expression of genes identified in our transcriptome analysis, mainly at exponential phase.

Among those genes downregulated in the *spdR* mutant, four had their expression increased in the wild type strain after entry into stationary phase (CC0445, CC0446, CC0583, and *cspD*) (Fig. 2), revealing a crucial role of SpdR in these growth phase inductions. The CC0583 and *cspD* genes were also previously shown to be induced at stationary phase in a DNA microarray assay [16]. Conversely, the other four downregulated genes either displayed no change in expression in the wild type strain at stationary phase (CC0517, CC0731 and *hfq*) or had the corresponding transcript levels decreased (CC1991). SpdR also plays a major contribution in regulating these genes at stationary phase, as judged by the lower expression in the *spdR* mutant relative to the wild type strain. Therefore, the relative importance of other regulatory system(s) for expression of these four genes seems to be reduced when wild type cells enter into stationary phase under the conditions examined. In regards to the gene upregulated in the absence of *spdR* (CC3654), the expression analysis showed that this effect occurs due to reduction in the transcript levels in the wild type strain at stationary phase, whereas no change is observed in the *spdR* mutant. Thus, this result suggests that SpdR is required for decreasing CC3654 expression at stationary phase.

A closer inspection of the newly identified SpdR-regulated genes revealed that a substantial percentage of these genes are predicted to encode proteins playing important roles in modulating gene expression. Among the regulatory genes, there are three (CC0445, CC3164 and CC3270) predicted to encode transcriptional regulators, two belonging to the GntR family, whose members act on diverse biological processes, and one to the Cro/CI family [35]. Most if not all transcriptional regulators from these families function as repressors, so it is conceivable to assume that downregulation of these genes in the *spdR* mutant could lead to increased expression of the five genes in the same strain according to microarray experiments. The GntR-type regulator encoded by CC0445 was previously characterized as NagR, and is located at the *nag* gene cluster that contains genes required for GlcNAc transport and metabolism [36], probably regulating the utilization of this carbon source.

The downregulated gene CC0653 is predicted to encode a CdnL ortholog, belonging to the large CarD_CdnL_TRCF family of bacterial RNA polymerase-interacting proteins. In contrast to CarD and TRCF from the same family, CdnL lacks a detectable DNA-binding domain [37]. CdnL proteins that had their function investigated were found to promote the formation of the open transcriptional complex, therefore stimulating transcription [38, 39]. All functionally characterized CdnL homologs proved to be essential for bacterial viability [40–42]. Furthermore, CdnL has been implicated in stress resistance, as depletion of the protein in *Mycobacterium tuberculosis* leads to sensitivity to oxidative stress, nutrient starvation and DNA damage [41], and the homologue in *Borrelia burgdorferi* is expressed exclusively at low temperature, a condition mimicking the bacteria within its arthropod vector [42]. Although no functional data is currently available for *C. crescentus* CC0653, the presumption that this gene is also required for viability of the bacterium [34] is in accordance to the role reported for other CdnL homologs. CC0653 was previously identified in a DNA microarray analysis as being 2-fold upregulated under iron limitation in a Fur-independent manner [43], indicating to have a role in response to nutrient limitation. Therefore, SpdR could indirectly activate transcription of a subset of genes in starvation conditions by means of the product of CC0653.

CC0035 and CC1247 are predicted to encode the ribosomal proteins S15 and S10, respectively, which are part of the smaller ribosome subunit. Although these genes belong to large operons, only these two genes were differentially expressed, indicating an additional level of regulation. Ribosomal protein S10, also called NusE, has a dual role in the cell, being also involved in transcriptional antitermination [44]. Interestingly, CC3205, predicted to encode NusG, another transcription antiterminator, also had its transcription altered. NusG is necessary for most Rho-mediated termination events in vivo [45, 46] and together with NusA, NusB and NusE promotes readthrough of terminators [44]. The C-terminal domain of NusG binds alternatively the transcriptional terminator Rho or NusE, coupling transcription to translation, so that transcription rates follow those of translation, adjusting the whole system to the nutritional needs of the cell [47–49]. This is particularly important if we consider that the rate of translation is also affected by the presence of secondary structures on mRNA [50]. The fact that both *nusE* and *nusG* were differentially expressed could indicate that SpdR mediates the control of transcription and translation rates at stationary phase. This idea agrees with the fact that it also activates expression of CspD, a protein with two putative RNA-binding domains that could have a role in preventing the formation of secondary structures on the mRNA.

The SpdR-dependent gene CC1745 is predicted to encode Hfq, a protein that helps small regulatory RNA to identify and anneal to their target mRNAs, and therefore is an important factor for global gene regulation. It was previously described for *E. coli* that the cold-shock proteins CspC and CspE, and Hfq positively regulate translation of the stationary sigma factor RpoS [51, 52], indicating that these RNA binding proteins can work in the same pathway of adaptation to stationary phase. Also downregulated in the absence of *spdR* and in the same transcriptional unit with CC1745, CC1746 is predicted to encode the protein HflX, one of the few members of the P-loop family of GTPases that are distributed throughout all domains of life [53]. Although its exact role remains undisclosed, HflX is currently regarded as a ribosome-associating protein [54–56]. This interaction stimulates GTP binding, GTPase activity and conformational change of HflX [57], all properties expected for a nucleotide-dependent molecular switch with a role in protein synthesis. Interestingly, *hflX* was not found to be an essential gene in *C. crescentus* [34], suggesting that the protein plays a more specialized function, and could have a role in responding and adapting to particular adverse conditions such as stationary growth phase. In this regard, HflX could be involved in the translation of a particular set of mRNAs or it may improve the efficiency of the protein synthesis machinery.

Also downregulated in the *spdR* mutant, CC0873 encodes toxin ParE1 of the toxin-antitoxin system ParD-ParE [58, 59]. The ParE toxin from *E. coli* plasmid RK2 has been described to inhibit DNA gyrase and thereby block DNA replication [60]. Crystallization studies of the ParD/ParE system encoded by the *C. crescentus* genes CC0873 and CC0874 showed that system forms an α_2_β_2_ heterotetramer in which ParD antitoxin helices bind to a conserved groove on the ParE toxin [58]. Expression of *parDE1* was shown to be induced by heat shock, but not in other stress conditions such as heavy metals, nitric oxide-induced oxidative stress or hypoxia [59]. Although the exact function of *C. crescentus* ParE1 remains to be investigated, it was demonstrated that overexpression of a C-terminal truncated ParE1 allele (ParE1(1–92)) caused loss of viability by inhibiting cell division, but did not affect cell growth [59]. Therefore, the finding that ParE1 expression is dependent on SpdR agrees with the role of toxins on bacterial adaptation to stationary phase. Interestingly, only the *parE* toxin gene was downregulated in the *spdR* mutant, although this gene is co-transcribed with *parD* [30], suggesting an additional regulatory mechanism to overcome the neutralizing effect of ParD1 antitoxin.

### Identification of direct targets of SpdR

This overrepresentation of genes encoding regulatory proteins in the SpdR regulon suggests that SpdR might not directly control expression of all genes identified by DNA microarray experiments. Instead, alteration in expression of at least some genes in the *spdR* mutant could be a consequence of downregulation of one or more SpdR-dependent transcriptional regulators.

In order to verify whether the SpdR protein directly regulates the expression of the genes identified in the transcriptome analysis, a search for regulatory sequences recognized by SpdR was performed using the RSAT platform (Regulatory Sequence Analysis Tools) [29]. The region from −300 to +200 relative to the putative translation start codon of each gene downregulated in the *spdR* mutant was screened for a sequence similar to that previously identified as the SpdR-binding motif of the *cspD* promoter region (CTGCGAC-N_5_-GTCGCGG) [21], allowing for up to two substitutions. This analysis revealed a putative sequence upstream of only two genes in addition to *cspD*, namely CC0517 and CC1746 (*hflX*). The sequence upstream of *hflX* is actually within the coding region of *hfq*, which is in the same transcriptional unit [30]. Interestingly, when a similar search was performed using a sequence better matching to a perfect palindromic motif (CTGCGAC-N_5_-GTCGCAG; A instead of G in the position underlined), the sequence upstream of *hflX* no longer fulfills the cutoff criteria, as three substitutions are needed with respect to the input motif. Using the other possibility to make the sequence upstream of *cspD* a perfect palindromic motif (CCGCGAC-N_5_-GTCGCGG; C instead of T in the position underlined), neither CC0517 nor CC1746 would have a SpdR-binding motif.

**Fig. 3:**
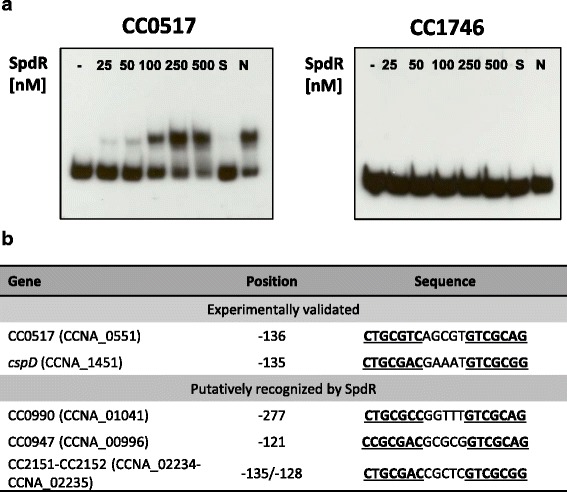
Analysis of SpdR binding motifs. **a** SpdR-binding assays to CC0517 and CC1746. DNA fragments containing the regions upstream of genes CC0517 and CC1746 were ^32^P-labeled and incubated with increasing concentrations of His_6_-SpdR (25, 50, 100, 250 and 500 nM) in an electrophoretic mobility shift assay (EMSA). As negative control, a reaction was carried out without His_6_-SpdR (−). In a competition assay, His_6_-SpdR was utilized at a 250 nM concentration and a 30x excess of unlabeled competitor fragment was added as follows: S, unlabeled specific fragment; N, unlabeled non-specific fragment. **b** Sequences recognized by the SpdR protein. An *in silico* search in *C. crescentus* NA1000 genome was performed with the consensus CTGCGAC-N_5_-GTCGCAG derived by the EMSA experiments. The ‘DNA pattern’ tool of RSA website [29] was used in the search, and one substitution was allowed. The position indicated refers to the first nucleotide of the sequence shown relative to the putative start codon in the NA1000 strain (+1). The same sequence is proposed to control expression of CC2151 and CC2152, which are divergently transcribed. The position of this sequence with respect to each gene is shown; for CC2152, the position refers to the nucleotide at the position 3’ of the sequence shown, which corresponds to the 5’ end of the reverse complementary sequence. Gene numbers refer to the CB15 strain (CC) and the correspondent number in NA1000 strain (CCNA)

To establish whether any of the newly identified sequences is truly a recognition motif of SpdR, EMSAs were performed using the purified recombinant His_6_-SpdR protein. Decreased mobility due to His_6_-SpdR binding was evident only for the fragment composed of the regulatory region of CC0517, but not for *hflX* (Fig. 3a). This finding therefore prompted us to suppose that the perfect palindromic motif CTGCGAC-N_5_-GTCGCAG is the best recognition sequence for SpdR, which deviates in one position in both CC0517 and *cspD* (Fig. 3b). Interestingly, when a search for the perfect palindromic SpdR-binding motif was carried out in the region from −300 to +200 relative to the putative translation start codon of all *C. crescentus* NA1000 genes (those not differentially expressed in the absence of *spdR* according to the transcriptome analysis), only three additional sequences deviating from the input motif in one position were identified (upstream of CC0947, CC0990, CC2151 and CC2152; the latter two genes are divergent from the same sequence) (Fig. 3b). This observation indicates that the SpdR-binding sequence is just occasionally found in the genome of *C. crescentus*. Therefore, these genes represent candidates to be SpdR-regulated, probably under a distinct condition from the one used in our assays that affects expression and/or activity of this regulatory protein. In fact, both CC0947 and CC2151 were upregulated 3.5-fold at stationary phase in a DNA microarray assay of stationary x exponential phase in PYE [16], and CC0990 also showed increased expression, but it did not fall within our cutoff criteria.

There is a possibility that SpdR may require additional factor(s) for binding to less conserved sites or even half-sites. However, a whole genome search for half the consensus sequence (CTGCGNC or CNGCGAC) was carried out, and produced too high a percentage of matches to be significant, probably due to the high GC content of the genome. These findings suggest that the probable SpdR-binding site requires a palindromic motif very similar to that found in *cspD* and CC0517.

### Effect of *cspD* and CC0517 on expression of SpdR-dependent genes

The restricted number of genes directly regulated by SpdR (*cspD* and CC0517) identified in this work is in agreement with the overrepresentation of regulatory proteins among the genes dependent on SpdR. The CspD protein has two Cold Shock Domains [14], suggesting it is a putative nucleic acid-binding protein, so it is reasonable to rationalize that it could affect the expression of some SpdR-dependent genes, especially those lacking an obvious motif for SpdR binding. Likewise, even though the function of CC0517 cannot be easily predicted from its deduced amino acid sequence, it could have some relevance for the expression of SpdR-regulated genes. In order to investigate this, a ∆CC0517 strain (MM80) was constructed and analyzed along with a *cspD* mutant [14] with respect to the expression of several SpdR-dependent genes at both exponential and stationary phases. The MM80 strain showed no obvious phenotype, presenting normal growth rate and showing no alterations in morphology or viability at stationary phase (Additional file 3: Figure S2). This is in agreement with the fact that neither the *spdR* nor the *cspD* mutants have a stationary phase phenotype [12, 14, 21].

**Fig. 4:**
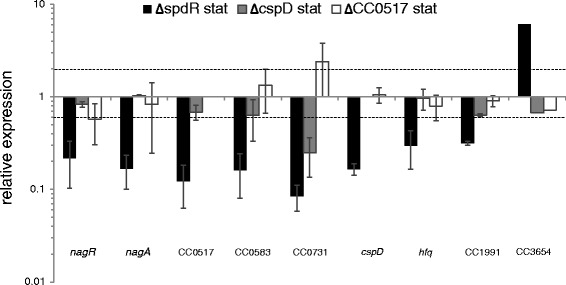
Expression of SpdR-regulated genes in *cspD* and CC0517 mutant strains. Expression of the indicated genes was analyzed by qRT-PCR using total RNA samples obtained from the wild type NA1000, ∆*spdR*, ∆*cspD and* ∆CC0517 strains at stationary growth phase. Results represent the expression of the corresponding gene in each mutant strain relative to wild type cells. Data represent mean values from two biological replicates, with bars indicating the standard errors

No differential expression of the SpdR-dependent genes analyzed was observed by comparing ∆CC0517 and Δ*cspD* to wild type at exponential growth phase (Additional file 4: Figure S1). However, CC0731 was found to be downregulated in the absence of *cspD* at stationary phase (Fig. 4). This result suggests that CspD is important for CC0731 expression at stationary phase, when SpdR and CspD are expected to play the greatest impact on gene expression. However, the magnitude of the decrease in CC0731 expression is lower when compared to that observed in cells lacking *spdR,* suggesting that another component in the SpdR network also contributes to the expression of this gene.

## Conclusion

This work has identified genes under control of the response regulator SpdR at stationary phase. The analysis of the putative roles of these genes suggests that the major aspects under SpdR regulation are transcription (mediated by regulators of the GntR, Cro and CdnL families, and NusE/NusG), the coupling of transcription and translation rates (mediated by NusE/NusG) and RNA metabolism (regulatory aspects mediated by Hfq, secondary structures putatively mediated by CspD). Interestingly, only two SpdR-dependent genes contained a sequence motif that is directly recognized by the response regulator. While it is uncertain whether one such gene (CC0517) contributes to the expression of SpdR targets, the involvement of *cspD* in the downstream effects of SpdR was demonstrated. Together, data presented here provide important insights into the regulatory network involving the response regulator SpdR and identified possible functions under its control, which are expected to contribute to the adaptation of *C. crescentus* to stationary phase.

## Availability of supporting data

The data sets supporting the results of this article are available in the Gene Expression Omnibus (GEO) repository, under accession number GSE71337 [http://www.ncbi.nlm.nih.gov/geo/query/acc.cgi?acc=GSE71337].

## Additional files

Additional file 1: Table S1. Bacterial strains, plasmids, and oligonucleotides used in this work. (PDF 296 kb) Additional file 2: The ARRIVE guidelines checklist. (PDF 471 kb) Additional file 3: Figure S2. Phenotypic analysis of the MM80 (ΔCC0517) strain. (PDF 369 kb) Additional file 4: Figure S1. Expression of SpdR-regulated genes in *cspD* and CC0517 mutant strains at exponential phase. (PDF 302 kb)

## Abbreviations

BCIP: 5-bromo-4-chloro-3'-indolyphosphate
cDNA: complementary DNA synthesized from RNA
Ct: cycle threshold
Cy3: Cyanine Dyes orange-fluorescent
Cy5: Cyanine Dyes far-red-fluorescent
GEO: Gene Expression Omnibus
His_6_: Hexahistidine tag
IPTG: isopropyl-beta-D-thiogalactopyranoside
NBT: nitro-blue tetrazolium
OD_600_: optical density at 600 nm
ORF: open reading frame
PAGE: Polyacrylamide Gel Electrophoresis
PCR: polymerase chain reaction
ROX: Dye designed to normalize the fluorescent reporter signal in real-time
rpm: rotations per minute
RT-PCR: reverse-transcription polymerase chain reaction
SDS: Sodium Dodecyl Sulfate
SYBR Green: asymmetrical cyanine dye
TBE: Tris/Borate/EDTA
TBS: Tris-buffered saline
WT: wild type
