# Supplementary material for: Transcriptomic analysis of the stationary phase response regulator SpdR in Caulobacter crescentus
Source: BMC Microbiol. 2016 Apr 12;16:66. doi: 10.1186/s12866-016-0682-y (PMC4830024; doi:10.1186/s12866-016-0682-y)
Supplement: Additional file 1: Table S1. — Bacterial strains, plasmids, and oligonucleotides used in this work. (PDF 296 kb) [file 12866_2016_682_MOESM1_ESM.pdf]

Supplemental Material

Carolina A. P. T. da Silva, Rogério F. Lourenço, Ricardo R. Mazzon, Rodolfo A. Ribeiro, and Marilis V. Marques. Transcriptomic analysis of the stationary phase response regulator SpdR in *Caulobacter crescentus*

Table S1. Bacterial strains, plasmids, and oligonucleotides used in this work

**Strain, plasmid or oligonucleotide**	**Description**	**Reference or source**[a]
Caulobacter crescentus		
NA1000	synchronizable derivative of CB15	Evinger and Agabian, 1977
MM80	NA1000 ΔCC0517	This Work
MM85	NA1000 Δ*spdR*	This Work
Δ*cspD*	NA1000 Δ*cspD*	Lang and Marques, 2004
SP0200	NA1000 Δ*spoT*	Da Silva et al., 2010
Escherichia coli		
DH5α	(Φ80*lacZ*ΔM15) *hsdR17 recA1 endA1 gyrA96 thi-1 relA1*	Hanahan, 1983
BL-21	*E. coli B F– dcm ompT hsdS(rB–mB–) gal*	Stratagene
S17-1	294::RP4-2(Tc::Mu)(Km::Tn7)	Simon et al., 1983
**Plasmids**		

pGEM	cloning vector, Ap^r	Promega
pET28a	expression vector, Kan^r	Novagen
pRK*lacZ*290	Reporter vector for transcriptional fusions to *lacZ,* Tet^r	Gober and Shapiro, 1992
pNPTS138	Suicide vector, containing *oriT sacB*, Kan^r	D. Alley, unpublished
pCA50	expression vector pET28a containing the *spdR* gene	This Work
pCA60	pRK*lacZ*290 with the *spdS* regulatory region	This Work
**Oligonucleotides**		
AUTO-1	CGAATTCCTTCGGCACATAGGGGGCCG	
HIST-1	CGGATCCGGCCGCAGAATTGTCCAGAAT	
HIP-1	GCCGGCTCAAGCTTTCGCCG	
HIP-2	GGGATCCCGATGCGGCGCGAGCGTTGG	
HIP-3	GGGATCCCAGAGCCTGACCCGCTTCCTC	
HIP-4	CGAATTCGCGCAGAAACTACGCCGTGC	
RR1	CGGGCCCCCAACTCCAATCTGCTGTGG	
RR2	TGGATCCTCCGCCATAAAAGTCAGCGC	
RR3	CGGATCCAGAAGCGTTTAAGTTGCTCCCTC	
RR4	AGAATTCGTTTGAATTATAATCGGGAGGA	
RT CC_0247 Forward	ATGCTGTTCTGGACATGCGGCT	

RT CC_0247 Reverse	TGTTGCCATAGCCCGTCAGCAT
RT CC_0445 Forward	TGGCGGACGAATTCAACATC
RT CC_0445 Reverse	AACGAGGACAGCTTGGAGAA
RT CC_0446 Forward	AGCCCTACAAGTCGAGCAAC
RT CC_0446 Reverse	CGCTGCAGGATGTAGTTGGA
RT CC_0517 Forward	TGTCTGAAGAGTACGCCAACG
RT CC_0517 Reverse	AAGCGCGTAGAGAAGGTCTG
RT CC_0583 Forward	TCAATTCGCAACTGGTGTCC
RT CC_0583 Reverse	CGCCAGGATCGTCTGAATCT
RT CC_0679 Forward	GCTCCGCTTTGATCTCTGCCTT
RT CC_0679 Reverse	TCGATCCGATGAACCCCAACCT
RT CC_0731 Forward	CCGCCAGAAGCGCGGCCGGG
RT CC_0731 Reverse	GTCGCTAGGCGAGCGCTCAT
RT CC_1387 Forward	AGGACGTGCTTCTGCATGTGAC
RT CC_1387 Reverse	AGATTGACGACCTCGCTGACCT
RT CC_1745 Forward	CGACAATTTCTGCGTCCTGC
RT CC_1745 Reverse	GCTCATAGAGCTGAACGGGT
RT CC_1991 Forward	CTGACACTGTCTCGCTGGAA
RT CC_1991 Reverse	CGGAAAGCTATCCAGCGTCT

RT CC_3654 Forward	TGAAAGGTCTGAAGGGCGTG
RT CC_3654 Reverse	AGCGGGAACATGTCGGGATA
RTCC_3098 Forward	AGGAAAAGTCGCTGCGGATGTC
RTCC_3098 Reverse	AGTTCTTCCAGGGTGGTCGGAT
SHIFT CC_0517 Forward	GCGCCTTGTCCCAGATCAG
SHIFT CC_0517 Reverse	ACAAGCGCGTAGAGAAGGTC
SHIFT CC_1746 Forward	TGACCTTGTTTGTGTGGGGG
SHIFT CC_1746 Reverse	ATGGATCACGAGCGCCTTC
M13 Forward (-20)	GTAAAACGACGGCCAG
M13 Reverse	CAGGAAACAGCTATGAC
REG-1	CGAATTCATGGCGGATATCGGAGAACT
REG-2	CTTCGAAGCGAGGGAGCAACTTAAACG

[a] References:

Lang EA, Marques MV. Identification and transcriptional control of Caulobacter crescentus genes encoding proteins containing a cold shock domain. J Bacteriol. 2004;186(17):5603–13

da Silva CA, Balhesteros H, Mazzon RR, Marques MV. SpdR, a response regulator required for stationary-phase induction of Caulobacter crescentus cspD. J Bacteriol. 2010;192(22):5991–6000.

Evinger M, Agabian N. Envelope-associated nucleoid from Caulobacter crescentus stalked and swarmer cells. J Bacteriol. 1977;132(1):294–301.

Hanahan D. Studies on transformation of Escherichia coli with plasmids. J Mol Biol. 1983;166(4):557–80.

Simon R, Priefer U, Puhler A. A broad host range mobilization system for in vivo genetic engineering: transposon mutagenesis in gram negative bacteria. Nat Biotechnol. 1983;1:784–91.

Gober JW, Shapiro L. A developmentally regulated Caulobacter flagellar promoter is activated by 3' enhancer and IHF binding elements. Mol Biol Cell. 1992;3(8):913–26.
