# Supplementary material for: Transcriptomic analysis of the stationary phase response regulator SpdR in Caulobacter crescentus
Source: BMC Microbiol. 2016 Apr 12;16:66. doi: 10.1186/s12866-016-0682-y (PMC4830024; doi:10.1186/s12866-016-0682-y)
Supplement: Additional file 3: Figure S2. — Phenotypic analysis of the MM80 (ΔCC0517) strain. (PDF 369 kb) [file 12866_2016_682_MOESM3_ESM.pdf]

Supplemental Material
Carolina A. P. T. da Silva, Rogério F. Lourenço, Ricardo R. Mazzon, Rodolfo A. Ribeiro, and Marilis V. Marques. Transcriptomic analysis of the stationary phase response regulator SpdR in Caulobacter crescentus

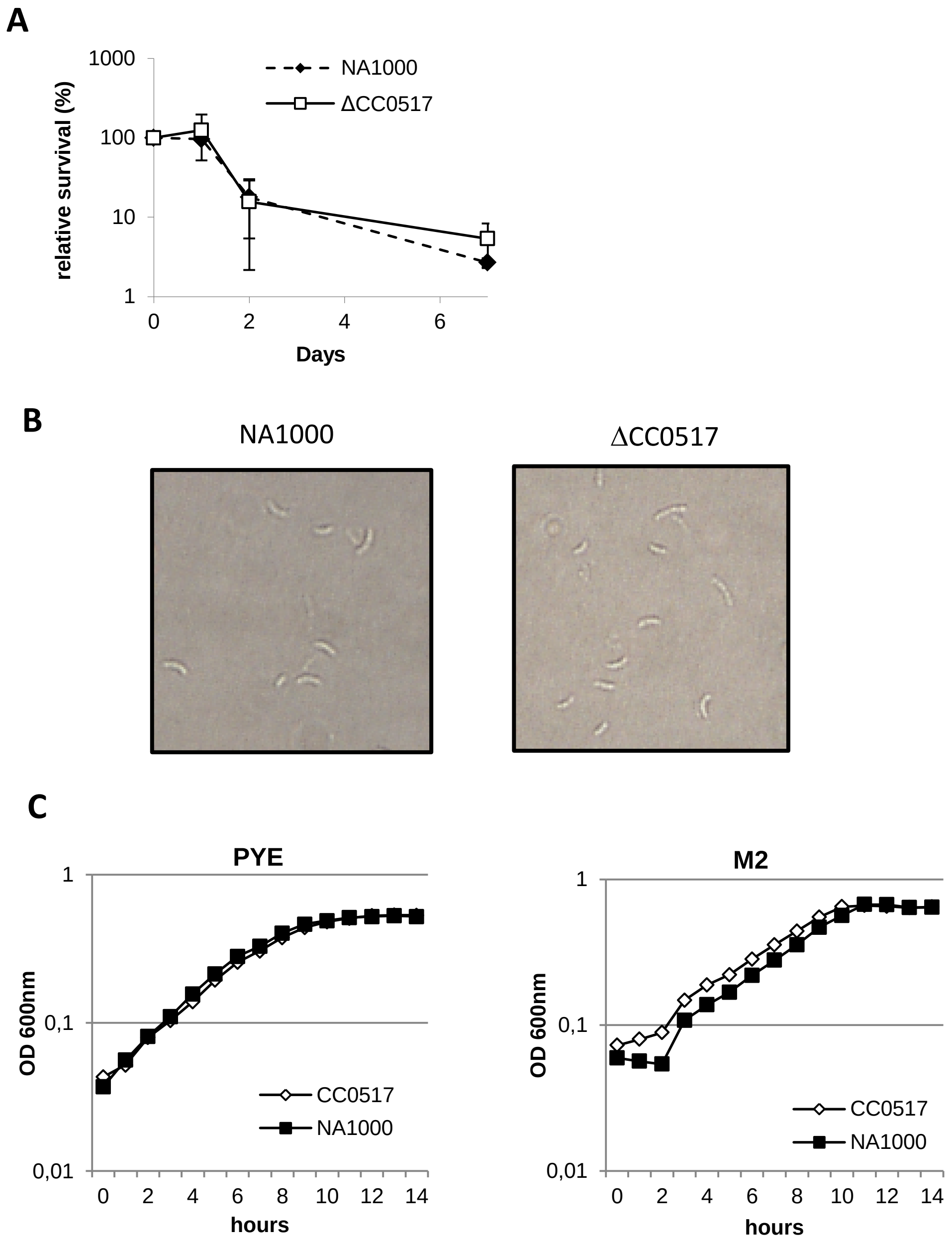

Figure S2. Phenotypic analysis of MM80 (ΔCC0517) strain. **A.** Viability assay of NA1000 and ΔCC0517 strains at stationary phase in PYE. CFU counts for each time point were determined and the percentage of viability is shown relative to CFU number in exponential phase for the respective strain (day zero). Data represent the means from three experiments in triplicate, with bars indicating the standard error. **B**. Morphological analysis of NA1000 and ΔCC0517 strains at early stationary phase (24h). **C**. Growth curves of NA1000 and ΔCC0517 strains in PYE and M2 media.
