# Supplementary material for: Transcriptomic analysis of the stationary phase response regulator SpdR in Caulobacter crescentus
Source: BMC Microbiol. 2016 Apr 12;16:66. doi: 10.1186/s12866-016-0682-y (PMC4830024; doi:10.1186/s12866-016-0682-y)
Supplement: Additional file 4: Figure S1. — Expression of SpdR-regulated genes in cspD and CC0517 mutant strains at exponential phase. (PDF 302 kb) [file 12866_2016_682_MOESM4_ESM.pdf]

Supplemental Material

Carolina A. P. T. da Silva, Rogério F. Lourenço, Ricardo R. Mazzon, Rodolfo A. Ribeiro, and Marilis V. Marques. Transcriptomic analysis of the stationary phase response regulator SpdR in Caulobacter crescentus

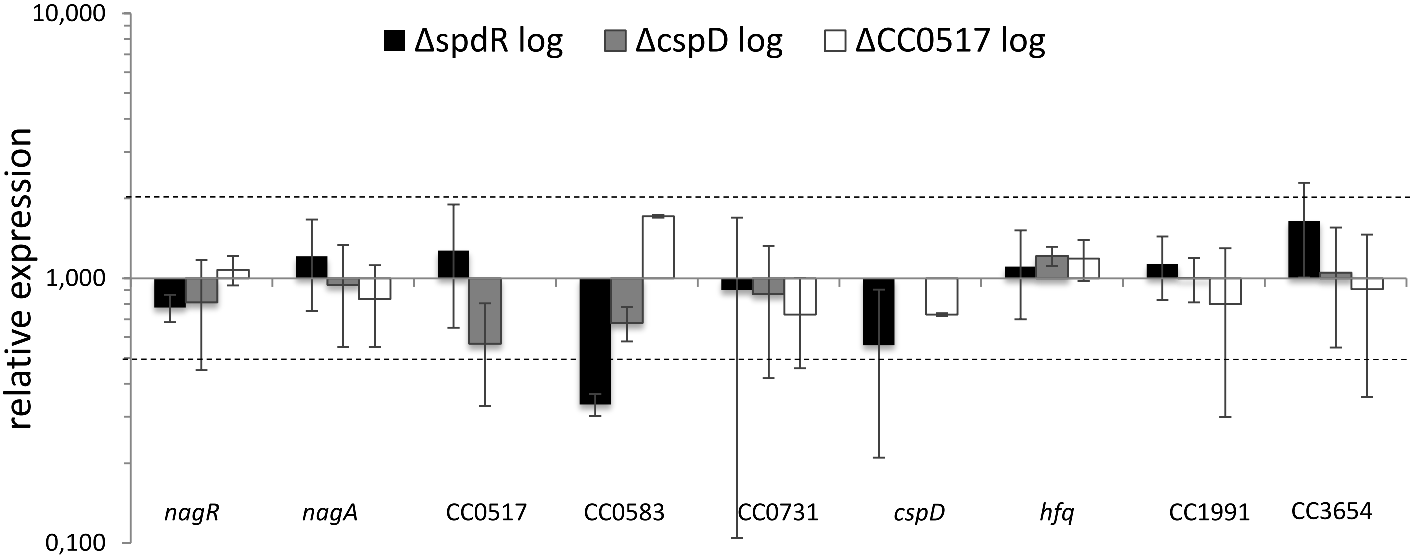

Figure S1. Expression of SpdR-regulated genes in *cspD* and CC0517 mutant strains at exponential phase. Expression of the indicated genes was analyzed by qRT-PCR using total RNA samples obtained from the wild type NA1000, Δ*spdR*, Δ*cspD and* ΔCC0517 strains at exponential growth phase. Results represent the expression of the corresponding gene in each mutant strain relative to wild type cells. Data represent means from two independent biological replicates, with bars indicating the standard error.
